# A Predictive Model for Midwives’ Compassion Fatigue Based on Stress Process Theory: Implications for Psychological Support and Human Resource Management

**DOI:** 10.1155/jonm/2656834

**Published:** 2026-08-31

**Authors:** Yian Zhan, Ying Li, Shan Yang

**Affiliations:** ^1^ The First Hospital of Lanzhou University, Donggang, Lanzhou Gansu, 730013, China, lzu.edu.cn; ^2^ School of Nursing, Lanzhou University, No. 28 Yanxi Road, Lanzhou Gansu, 730011, China, lzu.edu.cn

**Keywords:** compassion fatigue, midwives, predictive models, regression analysis

## Abstract

**Background:**

Compassion fatigue among midwives is prevalent and poses significant risks, adversely affecting both their personal well‐being and the quality of maternal and infant care. However, a tailored, practical tool for predicting compassion fatigue risk in this population is lacking.

**Objective:**

To analyze factors influencing compassion fatigue in midwives, construct a predictive model for compassion fatigue risk, and provide evidence‐based strategies for prevention.

**Methods:**

In this cross‐sectional study, 209 midwives from 29 public hospitals in western China were enrolled via convenience sampling. Compassion fatigue was assessed using the Professional Quality of Life Scale, alongside measures of the Traumatic Stress Scale for Midwives, Social Support Rating Scale, 10‐Item Connor–Davidson Resilience Scale, and Simplified Coping Style Questionnaire. Univariate analysis and multivariate logistic regression identified risk factors for compassion fatigue. A risk prediction model was constructed and a nomogram model was developed, followed by model evaluation and internal validation.

**Results:**

Six independent predictors were identified: age, sleep quality, perceived recognition in the past month, frequency of traumatic events, and positive/negative coping strategies. The model achieved an area under the receiver operating characteristic curve (area under the curve [AUC]) of 0.820 (95% confidence interval [CI]: 0.763–0.876). After internal validation via bootstrapping, the AUC decreased to 0.786 (95% CI: 0.779–0.815). The Hosmer–Lemeshow test yielded *χ* ^2^ = 5.777, *p* = 0.672.

**Conclusion:**

Based on the stress process theory, this study found that 91.86% of midwives experienced compassion fatigue at any level; 63.6% had moderate to severe levels—the threshold considered clinically significant for the purpose of the prediction model. Six independent predictors were identified: age, sleep quality, perceived recognition in the past month, frequency of traumatic events, and positive/negative coping. A nomogram prediction model was developed using these predictors. The model demonstrated good discriminative ability (AUC = 0.820, 95% CI: 0.763–0.876) and calibration (Hosmer–Lemeshow *p* = 0.672). This nomogram provides nursing managers with a practical, low‐cost tool to identify midwives at high risk of compassion fatigue, providing a reference for targeted preventive strategies.

**Implications for Nursing Management:**

This nomogram may assist managers in early identification of midwives at risk for compassion fatigue and facilitate risk‐stratified preventive strategies, with external validation needed before broader clinical implementation. The findings highlight the actionable areas for intervention—including psychological support, trauma‐informed care, sleep and workload management, and professional recognition practices—which may help mitigate compassion fatigue and promote workforce stability.

## 1. Introduction

Compassion fatigue is a professional psychological issue characterized by emotional exhaustion and diminished compassion resulting from prolonged work stress and has become a widespread challenge faced by healthcare workers globally [1]. Midwives, who serve as a core force safeguarding maternal and infant health, are particularly vulnerable due to their unique working environment. They provide one‐to‐one companionship throughout labor, requiring sustained, high‐intensity emotional labor to respond to the pain and anxiety of parturient women [2]. Concurrently, frequent exposure to traumatic childbirth events—including neonatal asphyxia, shoulder dystocia, and postpartum hemorrhage—places midwives at high risk for compassion fatigue [3, 4]. Indeed, 85.4% of midwives reported experiencing traumatic birth events, underscoring the pervasive nature of occupational trauma exposure within this specialty [3].

Studies indicate that the prevalence of moderate to severe compassion fatigue among midwives ranges from 73.9% to over 80% [5–7], which is considerably higher than rates reported among general clinical nurses [8]. Compassion fatigue not only damages midwives’ physical and mental health but also impairs their clinical judgment, reduces the quality of intrapartum care, and threatens maternal and neonatal safety [9, 10]. Prolonged compassion fatigue causes midwives to lose enthusiasm and motivation for their work, hindering their professional growth and career development, and consequently impacting the sustainable development of the entire midwifery workforce. This situation is particularly pronounced in low‐ and middle‐income countries [11, 12]. The International Confederation of Midwives emphasizes that improving midwives’ retention is a core responsibility of administrators [13]. Therefore, addressing and effectively mitigating the risk of compassion fatigue among midwives is not only essential for safeguarding the physical and mental well‐being of individual midwives but also a critical management strategy for ensuring global maternal and newborn health, optimizing the allocation of healthcare resources, and achieving the Sustainable Development Goals.

The causes of compassion fatigue among midwives are complex and multifaceted [6, 7, 14–17], and the existing compassion fatigue assessment tools are predominantly universal scales that fail to specifically highlight and reflect the unique risks inherent in midwifery practice. Given the multifactorial etiology, a prediction model that integrates multiple risk factors may offer a more effective approach for identifying high‐risk individuals in a timely manner. Xie et al. [18] developed a nomogram‐based prediction model for emergency department nurses, demonstrating good calibration and clinical utility. However, compared with other nursing populations, midwives practice in a distinctly different occupational context—one characterized by sustained emotional engagement and repeated exposure to traumatic childbirth events—which may predispose them to a different risk pattern for compassion fatigue. Despite this specificity, no prediction model has yet been developed specifically for the midwife population. Therefore, there is an urgent need to develop compassion fatigue assessment tools specifically tailored for midwives. This would enable accurate and efficient identification of compassion fatigue among midwives, providing managers with insights into the current state of compassion fatigue within their workforce. Such tools would facilitate the development of effective, targeted management strategies, and corresponding intervention measures, thereby effectively reducing the incidence of compassion fatigue among midwives.

**FIGURE 1:**
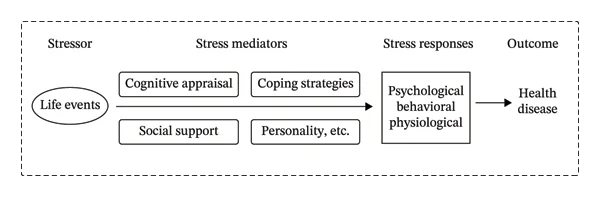
Theoretical model of the stress process (adapted from Wu and Jiang).

**FIGURE 2:**
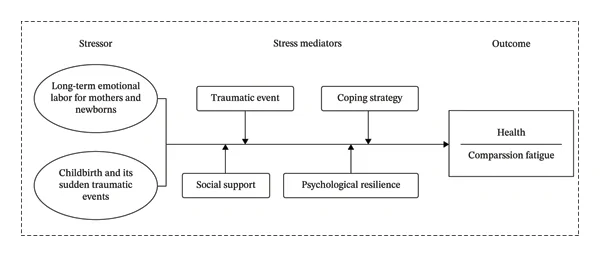
Multifactor model framework for midwives’ compassion fatigue.

This study references Wu and Jiang’s [19] theoretical model of the stress process (Figure 1), which shares core logical commonalities with Folkman et al.’s [20] cognitive appraisal theory. Both emphasize the dynamic process whereby stressors, through mediators, lead to adaptive outcomes. The model posits that after stressors act upon an individual, corresponding adaptive outcomes emerge through the interaction of stress mediators. It specifically highlights several important and common stress mediators, such as coping strategies, social support, and personality. This model provides a theoretical framework for the development of compassion fatigue. Midwives’ prolonged exposure to sustained, high‐intensity stressors—specifically the demands of managing childbirth and its sudden traumatic events—interacts with stress mediators to produce corresponding stress responses, culminating in either the emergence or absence of compassion fatigue. To systematically apply the stress process theory to the present study, the selected variables were operationalized to correspond directly to the key theoretical components. Specifically, within our adapted framework, the stressors are defined as exposure to traumatic childbirth events. The stress mediators are operationalized as traumatic cognition (representing cognitive appraisal of the stressor) [21, 22], coping strategies (including both positive and negative coping) [23], social support (encompassing support from colleagues, family, and friends) [6, 14], and psychological resilience (representing the personality dimension most relevant to adversity adaptation in this context) [24]. The stress response outcome is operationalized as the level of compassion fatigue experienced by the midwife. Drawing upon this clear theoretical mapping, we developed the theoretical model for this study—the multifactorial compassion fatigue model (Figure 2)—to guide the selection of survey indicators in the cross‐sectional research.

Based on the stress process theory, this study employed a cross‐sectional design to investigate the determinants of compassion fatigue in midwives and established a visualized risk prediction model tailored for midwives. This aims to assist clinical managers quickly and easily screen high‐risk individuals for compassion fatigue, providing a reference basis for implementing targeted, preventive management measures at an early stage.

## 2. Materials and Methods

### 2.1. Study Design and Participants

A multicenter, cross‐sectional study design was employed. Western China exhibits uneven socioeconomic development, with a multitiered healthcare system structure—a pattern mirroring that of many low‐ and middle‐income countries. Based on this context, this multicenter study encompassed 29 healthcare institutions of varying levels across different regions of western China. By focusing on western China, the research aims to provide insights for regions worldwide facing similar challenges.

The participants included midwives based on the following inclusion criteria: possession of a midwifery practice license; actively working as a midwife during the survey period (not on extended leave); midwifery practice duration > 1 year; voluntary participation in the study; and signing of informed consent. The exclusion criteria were as follows: history of psychiatric disorders; use of psychiatric medications within 1 month before the survey; and withdrawal from the study. This cross‐sectional study adopted formula *N* = $Z_{\alpha /2}^{2}$ × *P* × (1 − *P*)/*d* ^2^ for sample size estimation. Based on a previous report [7], the prevalence of compassion fatigue among midwives was 90.96%. With *α* = 0.05 (*Z*_*α*/2_ = 1.96) and *d* = 0.05, the calculated minimum sample size was 127 participants. After accounting for a 10% rate of invalid questionnaires, the required sample size was determined to be 141 participants.

### 2.2. Data Collection and Ethical Consideration

This study was approved by the Institutional Review Board of the researchers’ affiliated university (LZUHLXY20250053). Data collection took place between September 16 and 30, 2025. Researchers explained the study’s purpose, methods, and procedures to the nursing departments of each of the 29 hospitals in this study. Following institutional approval, an anonymous electronic questionnaire link was forwarded to midwives by nursing administrators solely for the purpose of dissemination. Nursing administrators were responsible only for forwarding the link and facilitating the distribution process; they were not granted back‐end access privileges to view individual responses or real‐time completion status. The questionnaire was designed without any personal identifiers, such as names or employee IDs, and the survey platform’s tracking and autocapture functions were disabled to prevent the automatic collection of IP addresses or other potentially identifying metadata. The cover page of the questionnaire explicitly stated the study’s purpose, significance, and confidentiality protections and informed participants that their involvement was entirely voluntary and that they could withdraw at any time without any adverse consequences. After the survey closed, two researchers independently screened the submissions for completeness, and questionnaires with substantial missing data were excluded from analysis. No individual‐level data were accessible to hospital administrators at any stage of the study.

### 2.3. Measurements

The integrated theoretical framework in this study guided the selection of research variables and assessment tools, primarily encompassing five dimensions: compassion fatigue (Professional Quality of Life Scale), traumatic events (Traumatic Stress Scale for Midwives), social support (Social Support Rating Scale), psychological resilience (10‐Item Connor–Davidson Resilience Scale), and coping strategies (Simplified Coping Style Questionnaire). Concurrently, we investigated the demographic data and occupational characteristics of the research subjects. For some scales, we selected translated and adapted Chinese versions to enhance participants’ comprehension.

#### 2.3.1. Demographic Data and Occupational Characteristic Questionnaire

This questionnaire was developed based on a review of prior literature and validated through focus group discussions and clinical practice. It aims to systematically collect demographic data and occupational characteristics of the study subjects. Demographic data include age, marital status, years of employment, hospital level, educational level, professional title, monthly income, form of employment, sleep quality, number of children, children’s status, and number of adverse deliveries. Occupational characteristics include night shift frequency, job satisfaction with compensation, workload, shift duration, satisfaction with teamwork and work atmosphere, annual leave entitlement, and perceived recognition in the past month.

#### 2.3.2. Professional Quality of Life Scale

The Professional Quality of Life Scale was developed by Stamm et al. in 2005 to measure compassion fatigue among research subjects [25]. In 2013, Chinese scholars Chen and Wang [26] translated, adapted, and revised the original scale to create the Chinese version of the Compassion Fatigue Scale, incorporating Chinese cultural contexts and national conditions. The scale comprises 30 items across three dimensions—compassion satisfaction, burnout, and secondary traumatic stress—with 10 items per dimension. Items are rated on a five‐point Likert scale, ranging from 1 (*strongly disagree*) to 5 (*strongly agree*). Items 1, 4, 15, 17, and 29 are reverse‐scored. The severity of compassion fatigue was determined based on established cutoff scores for each of the three dimensions: compassion satisfaction (< 37), burnout (> 27), and secondary traumatic stress (> 17). Specifically, if none of the three dimension scores exceeded their respective cutoffs, compassion fatigue was considered absent. Mild compassion fatigue was indicated if any one dimension score exceeded its cutoff. Moderate compassion fatigue was diagnosed when any two dimension scores surpassed their cutoffs. Severe compassion fatigue was defined by all three dimension scores exceeding their respective thresholds. This scale demonstrates good reliability and validity among Chinese clinical nurses, with a Cronbach’s *α* coefficient of 0.91. Cronbach’s *α* coefficients for the three dimensions are 0.87, 0.73, and 0.84, respectively. In the present study, after appropriate reverse‐scoring of relevant items, the total scale Cronbach’s *α* was 0.679, and the subscale *α* values were 0.905 (Compassion Satisfaction), 0.643 (Burnout), and 0.771 (Secondary Traumatic Stress). After excluding the five reverse‐scored items, the total scale *α* was 0.818 and the burnout subscale *α* was 0.751.

To identify midwives with clinically significant compassion fatigue, participants were categorized into a no/mild versus a moderate/severe groups based on clinical relevance. Moderate to severe compassion fatigue typically reflects symptoms that may impair professional functioning and patient care quality and require managerial or psychological intervention, whereas mild symptoms may represent subclinical or transient responses. A similar categorical approach was also used by Xin et al. [6] in their study of compassion fatigue among midwives.

#### 2.3.3. Traumatic Stress Scale for Midwives

The Traumatic Stress Scale for Midwives was developed by Kubota and Horiuchi [4] in 2022 to measure the frequency and impact of traumatic stress events experienced by midwives. Due to the differences in healthcare environments and cultures, Chinese researcher Pu et al. [27] adapted it cross‐culturally by removing Item 12 and modifying certain phrasing to enhance readability for study participants. This unidimensional scale comprises 15 items organized into two subscales: occurrence frequency and impact severity. Each item uses a four‐point Likert scale. Scores of 0–3 for occurrence frequency and impact severity correspond to “never occurred” to “frequently occurred” and “no impact” to “significant impact,” respectively. The scale does not calculate a total score. Both subscales range from 0 to 45 points, with higher scores indicating greater frequency of traumatic stress events experienced by midwives and more severe physical and psychological impact. This scale demonstrates good reliability and validity among Chinese midwives, with Cronbach’s *α* coefficients of 0.89 and 0.95 for the two subscales, respectively, and is thus considered appropriate for assessing midwives’ levels of traumatic stress. In the present study, Cronbach’s *α* coefficients for the two subscales were 0.841 and 0.949, respectively.

#### 2.3.4. Social Support Rating Scale

The Social Support Rating Scale was developed by Xiao [28] in 1986 to measure the degree of social support received by individuals. The scale comprises 10 items across three dimensions: objective support, subjective support, and utilization of support. Item 5 is subdivided into four sections (A, B, C, and D) for total scoring, with each section rated from 1 (None) to 4 (Full Support); Items 6 and 7 assign 0 points for “no sources” and corresponding points for “specific sources” based on the number selected, ranging from 0 to 9 points. Other items assign 1, 2, 3, or 4 points for selecting 1, 2, 3, or 4 options, respectively. A total scale score of < 33 indicates low social support, 33–45 indicates moderate social support, and > 45 indicates high social support. Higher total scores reflect greater social support. According to the original literature, the scale demonstrated good stability with a 2‐month test–retest reliability of 0.92. This scale demonstrates good reliability and validity across multiple studies and can be used to assess midwives’ social support levels. In this study, item analysis was conducted using the corrected item‐total correlation (CITC). The CITC values for the 10 items ranged from 0.275 to 0.576. All items, except item 2, exhibited correlation coefficients above 0.30, indicating good consistency between individual items and the overall construct of the scale.

#### 2.3.5. 10‐Item Connor–Davidson Resilience Scale

The 10‐item Connor–Davidson Resilience Scale was revised by Campbell‐Sills et al. [29] based on the 25‐item Connor–Davidson Resilience Scale developed by Connor and Davidson [30] in 2003. Chinese researchers Wang et al. [31] adapted it through cross‐cultural adjustments and reliability/validity testing to create the Simplified Psychological Resilience Scale. This 10‐item scale employs a five‐point Likert scale, with responses ranging from 0 (almost never) to 4 (always). Total scores range from 0 to 40, with higher scores indicating greater psychological resilience. The scale demonstrated a Cronbach’s *α* coefficient of 0.91 and a test–retest reliability coefficient of 0.90. Given its demonstrated good reliability and validity across multiple studies, it was considered suitable for assessing midwives’ psychological resilience levels in the present study. In the present study, Cronbach’s *α* coefficient for the total scale was 0.948.

#### 2.3.6. Simplified Coping Style Questionnaire

The Simplified Coping Style Questionnaire, developed by Xie [32] in 1998 based on a synthesis of relevant coping theory, assesses the attitudes and approaches research subjects may adopt when facing setbacks or difficulties. This 20‐item scale comprises two dimensions: positive coping (12 items) and negative coping (8 items). It employs a four‐point Likert scale: 0, 1, 2, and 3 represent “never,” “rarely,” “sometimes,” and “often,” respectively. A coping tendency score greater than 0 indicates that the respondent primarily uses positive coping strategies under stress, while a score less than 0 suggests a tendency toward negative coping strategies. The scale demonstrated good internal consistency reliability (Cronbach’s *α* = 0.90) and test–retest reliability (Cronbach’s *α* = 0.89). The positive coping subscale showed an internal consistency reliability of 0.89, while the negative coping subscale had a reliability of 0.78. Given its established validity and reliability for assessing individuals’ coping strategies, it was considered suitable for evaluating midwives’ coping strategies in the present study. In the present study, Cronbach’s *α* coefficient for the total scale was 0.924, and for the positive coping and negative coping subscales, it was 0.932 and 0.828, respectively.

### 2.4. Statistical Analysis

Data were entered by two operators using MS Excel software and cross‐checked repeatedly for accuracy. Analysis was performed using SPSS 26.0 and R 4.5.0 statistical software. The count data were described using frequency and percentage, with group comparisons performed using chi‐square tests. For continuous data meeting normal distribution, means ± standard deviation were used for description, and independent samples *t* tests were applied for group comparisons. For non‐normally distributed data, the median and interquartile range were used for description, with nonparametric Mann–Whitney *U* tests for group comparisons. Binary and ordinal variables were analyzed using chi‐square tests. Statistical significance was set at *p* < 0.05. Cases with missing data were excluded from the respective analyses. After univariate analysis of midwives’ general characteristics and risk factors associated with compassion fatigue, the comprehensive model coefficient and goodness‐of‐fit tests were conducted. After univariate analysis, all candidate variables were retained for multivariate analysis to avoid omitting important predictors regardless of their univariate significance, before fitting the final model, multicollinearity among independent variables was examined using the variance inflation factor (VIF), with a threshold of 10 indicating the presence of serious multicollinearity. All variables were included in a binary logistic regression analysis to identify independent influencing factors (*p* < 0.05). Using R software and its rms package, risk prediction models were constructed by incorporating risk factors identified through binary logistic regression into regression plots. Model performance was evaluated using the area under the curve (AUC) of the receiver operating characteristic (ROC) curve, Hosmer–Lemeshow goodness‐of‐fit test, and decision curve analysis. Internal validation was conducted via bootstrap sampling, with calibration curves plotted to assess model consistency. *p* < 0.05 was considered statistically significant.

## 3. Results

### 3.1. General Characteristics of Midwives and Univariate Analysis of Compassion Fatigue

This study employed convenience sampling to recruit midwives from 29 public hospitals in western China between September 16 and 30, 2025. A total of 227 questionnaires were collected. After excluding 18 invalid questionnaires (including incomplete responses and patterned responding), 209 valid questionnaires were retained, yielding an effective response rate of 92.07%. Among them, 124 (59.4%) were from large general hospitals, 37 (17.7%) from large specialized hospitals, 17 (8.1%) from medium‐sized general hospitals, and 31 (14.8%) from medium‐sized specialized hospitals. The mean Compassion Fatigue Scale score was (48.15 ± 9.50). Among them, 17 (8.1%) had no compassion fatigue, 59 (28.2%) had mild compassion fatigue, 105 (50.2%) had moderate compassion fatigue, and 28 (13.4%) had severe compassion fatigue. The overall prevalence rate of compassion fatigue was 91.86%. For the purpose of distinguishing midwives with clinically significant compassion fatigue from those with minimal or subclinical symptoms, participants were dichotomized into a no/mild compassion fatigue group (*n* = 76, 36.4%) and a moderate/severe compassion fatigue group (*n* = 133, 63.6%). Univariate analysis revealed 15 statistically significant variables (*p* < 0.05): age, years of service, professional title, sleep quality, number of children, children’s status, compensation, teamwork, annual leave duration, work atmosphere, perceived recognition in the past month, frequency of traumatic events, social support, psychological resilience, and positive coping (Table 1; full details are available in Table A1).

**TABLE 1:** General characteristics of midwives and univariate analysis results for compassion fatigue (*n* = 209).

Variable		Level of compassion fatigue		Statistics	*p*
		No or mild	Moderate and above		
Age, *n* (%)	20–30 years	14 (18.4)	45 (33.8)	3.693^2^	< 0.001^∗^
	31–35 years	22 (28.9)	53 (39.8)		
	36–45 years	33 (43.4)	30 (22.6)		
	> 45 years	7 (9.2)	5 (3.8)		

Years of employment, *n* (%)	1–5 years	15 (19.7)	33 (24.8)	2.236^2^	0.025^∗^
	6–10 years	22 (28.9)	50 (37.6)		
	11–15 years	16 (21.1)	32 (24.1)		
	> 15 years	23 (30.3)	18 (13.5)		

Professional title, *n* (%)	Junior nurse	3 (3.9)	15 (11.3)	2.317^2^	0.021^∗^
	Staff nurse	29 (38.2)	61 (45.9)		
	Charge nurse	40 (52.6)	52 (39.1)		
	Nurse manager or higher	4 (5.3)	5 (3.8)		

Sleep quality, *n* (%)	Normal	18 (23.7)	12 (9.0)	3.750^2^	< 0.001^∗^
	Occasional insomnia	38 (50.0)	55 (41.4)		
	Frequent insomnia	20 (26.3)	66 (49.6)		

Number of children, *n* (%)	0	13 (17.1)	32 (24.1)	2.288^2^	0.022^∗^
	1	26 (34.2)	59 (44.4)		
	2 or more	37 (48.7)	42 (31.6)		

Children’s status (Children in junior high school or above), *n* (%)	No	56 (73.7)	115 (86.5)	5.312^1^	0.021^∗^
	Yes	20 (26.3)	18 (13.5)		

Job satisfaction with compensation, *n* (%)	Satisfied	39 (51.3)	39 (29.3)	2.935^2^	0.003^∗^
	Normal	29 (38.2)	73 (54.9)		
	Unsatisfied	8 (10.5)	21 (15.8)		

Teamwork satisfaction, *n* (%)	Satisfied	47 (61.8)	47 (35.3)	3.600^2^	< 0.001^∗^
	Normal	24 (31.6)	69 (51.9)		
	Unsatisfied	5 (6.6)	17 (12.8)		

Annual leave entitlement, *n* (%)	≤ 4 days	7 (9.2)	25 (18.8)	2.868^2^	0.004^∗^
	5–9 days	34 (44.7)	71 (53.4)		
	≥ 10 days	35 (46.1)	37 (27.8)		

Work atmosphere satisfaction, *n* (%)	Satisfied	58 (76.3)	73 (54.9)	3.180^2^	0.001^∗^
	Normal	18 (23.7)	55 (41.4)		
	Unsatisfied	0 (0.0)	5 (3.8)		

Perceived recognition in the past month, *n* (%)	No	11 (14.5)	49 (36.8)	11.823^1^	< 0.001^∗^
	Yes	65 (85.5)	84 (63.2)		

Frequency of traumatic events, mean ± SD		8.05 ± 3.98	9.74 ± 4.71	2.662^2^	0.008^∗^

Social support, mean ± SD		44.75 ± 8.09	39.36 ± 7.84	4.724^3^	< 0.001^∗^

Psychological resilience, mean ± SD		27.50 ± 6.81	20.79 ± 7.71	5.916^2^	< 0.001^∗^

Positive coping strategies, mean ± SD		25.70 ± 6.72	19.82 ± 7.49	5.262^2^	< 0.001^∗^

Abbreviation: SD, standard deviation.

^1^ *χ* ^2^.

^2^ *Z*.

^3^ *t*.

^∗^ *p* < 0.05.

### 3.2. Multifactorial Analysis of Compassion Fatigue Among Midwives

After comprehensive model coefficient testing and goodness‐of‐fit testing (Tables A2), variables deemed statistically insignificant in univariate analysis were not arbitrarily discarded. All variables were ultimately sequentially incorporated into the logistic regression model using backward elimination (variable assignments are detailed in Table A3). Multicollinearity assessment showed that all VIF values were below the threshold of 10, indicating no serious multicollinearity (range: 1.350–7.829).

According to the results in Table 2, age 36–45 years (odds ratio [ORs] = 0.244, 95% confidence interval [CI] = 0.076–0.778), age > 45 years (OR = 0.082, 95% CI = 0.011–0.620), frequent insomnia (OR = 3.321, 95% CI = 1.118–9.869), perceived recognition (OR = 0.363, 95% CI = 0.155–0.853), and frequency of traumatic events (OR = 1.529, 95% CI = 1.044–2.240), positive coping (OR = 0.313, 95% CI = 0.194–0.506), and negative coping (OR = 1.874, 95% CI = 1.231–2.853) were factors influencing midwives’ compassion fatigue (*p* < 0.05).

**TABLE 2:** Multivariate logistic regression analysis results (*n* = 209).

Predictors	*B*	Se	Wald *χ* ^2^	*p*	OR	95% CI
Constant	2.226	0.938	5.630	0.018	—	—
Age (Ref: 20–30 years old)	—	—	—	—	1	—
31–35 years old	−0.188	0.514	0.134	0.715	0.829	0.303–2.269
36–45 years old	−1.411	0.592	5.678	0.017^∗^	0.244	0.076–0.778
> 45 years old	−2.499	1.031	5.870	0.015^∗^	0.082	0.011–0.620
Professional title (Ref.: junior nurse)	—	—	—	—	1	—
Staff nurse	−1.205	0.794	2.305	0.129	0.300	0.063–1.420
Charge nurse	−0.871	0.859	1.028	0.311	0.418	0.078–2.255
Nurse manager or higher	1.203	1.222	0.969	0.325	3.331	0.304–36.533
Sleep quality (Ref.: normal)	—	—	—	—	1	—
Occasional insomnia	0.631	0.522	1.460	0.227	1.879	0.675–5.229
Frequent insomnia	1.200	0.556	4.666	0.031^∗^	3.321	1.118–9.869
Perceived recognition (Ref.: no)	—	—	—	—	1	—
Yes	−1.013	0.436	5.411	0.020^∗^	0.363	0.155–0.853
Frequency of traumatic events	0.425	0.195	4.761	0.029^∗^	1.529	1.044–2.240
Positive coping strategies	−1.162	0.245	22.449	< 0.001^∗∗^	0.313	0.194–0.506
Negative coping strategies	0.628	0.214	8.581	0.003^∗∗^	1.874	1.231–2.853

*Note:* B, regression coefficient; p, probability.

Abbreviations: CI, confidence interval; OR, odds ratio; SE, standard error.

^∗^ *p* < 0.05.

^∗∗^ *p* < 0.01.

### 3.3. Development of a Predictive Model for Midwives’ Compassion Fatigue Risk

**FIGURE 3:**
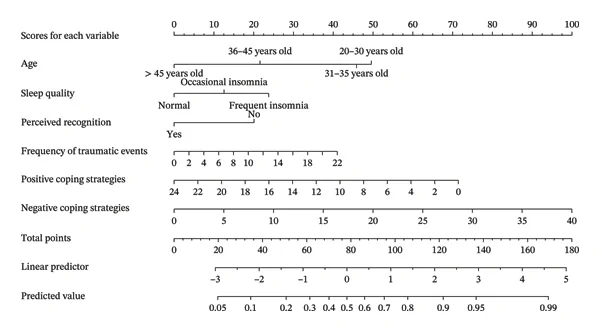
Nomogram prediction model for compassion fatigue in midwives.

Based on the results of the multivariate logistic regression analysis, this study utilized the rms package in R software to construct a nomogram prediction model for midwives’ compassion fatigue (Figure 3). Using the factors influencing midwives’ compassion fatigue levels as independent variables, the lrm() function was employed to construct a logistic regression model for the risk of compassion fatigue occurrence:

(1) LogitP=2.2261.4112.4991.2001.0130.4251.1620.628−×age=3645− years−×age>45+×frequent insomnia−×perceived recognition in the past month+×frequency of traumatic events−×positive coping+×negative coping.

The final model included six predictor constructs with a total of 9 estimated parameters. Accordingly, the events‐per‐variable (EPV) ratio was calculated as 76/9 ≈ 8.4, which is slightly below the commonly recommended minimum threshold of 10 [33].

The nomogram() function converts the partial regression coefficients of each variable in the model into visual scores. The fun parameter is set to plogis, mapping the model’s linear predicted values to the probability of experiencing compassion fatigue. The nomogram is designed for midwife managers as a screening tool to identify high‐risk individuals early. Each variable corresponds to a scale line, with values matching the scores above (Table A4). The total score is the sum of scores across variables, and the final predicted value on the risk probability axis represents the predicted probability of midwife compassion fatigue (Table A5).

### 3.4. Evaluation and Development of a Predictive Model for Midwives’ Compassion Fatigue Risk

**FIGURE 4:**
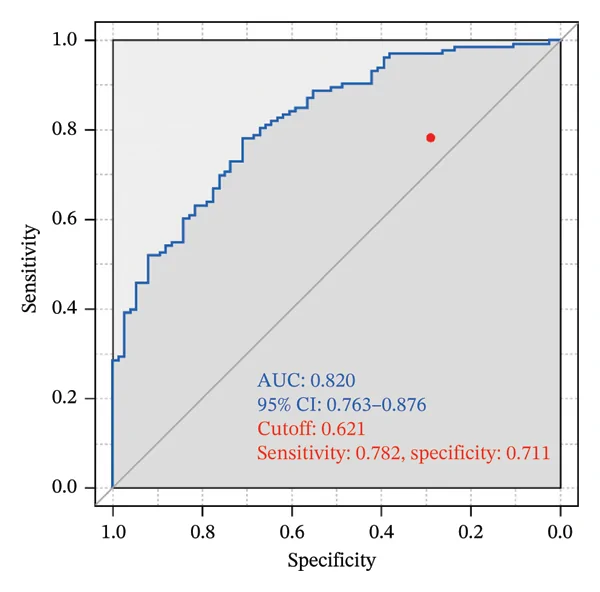
Receiver operating characteristic curve for the nomogram prediction model. Abbreviations: AUC, area under the curve; CI, confidence interval.

The ROC curve was plotted using the pROC package in R software to evaluate the model’s discriminatory ability. The ROC curve calculates sensitivity and specificity at different thresholds by varying the predicted probability threshold and then connects these points to form a curve. The AUC quantifies the model’s discriminative capability, with AUC > 0.7 indicating acceptable discrimination and > 0.8 indicating good discrimination. The results showed that the model achieved an AUC of 0.820 (95% CI: 0.763–0.876) with an optimal cutoff value of 0.621. At this threshold, the specificity was 0.711 and sensitivity was 0.782, indicating good model discrimination and predictive accuracy (Figure 4).

**FIGURE 5:**
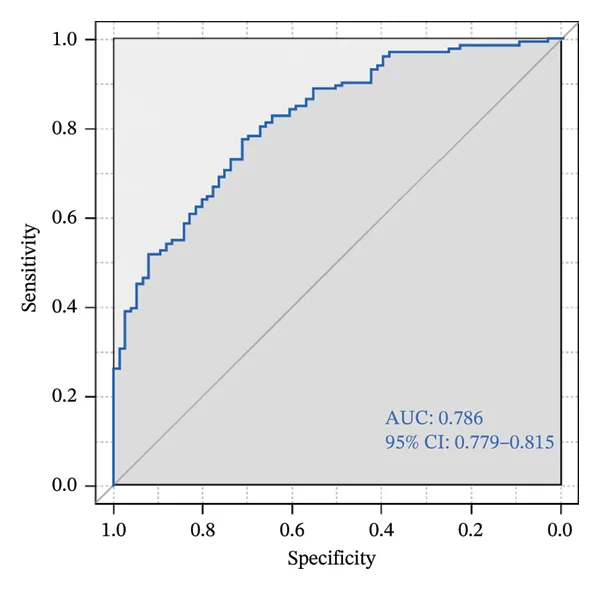
Receiver operating characteristic curve for the internal validation of the nomogram prediction model. Abbreviations: AUC, area under the curve; CI, confidence interval.

Internal validation was performed using the rms package, employing 1000 resampled bootstrap samples with replacement to assess model stability and generalization ability on new samples. After 1000 resamples, the model coefficients stabilized (Table A6), with a calibrated AUC of 0.786 (95% CI: 0.779–0.815), indicating good discriminant consistency (Figure 5).

**FIGURE 6:**
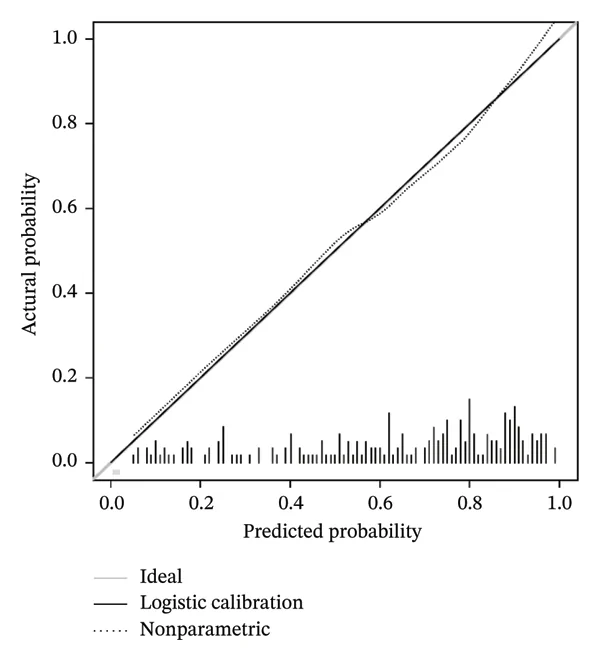
Calibration curve of the nomogram prediction model.

Model calibration was further assessed using the calibration curve and the Hosmer–Lemeshow goodness‐of‐fit test. The calibration curve was plotted by comparing predicted mean proportions with observed proportions, ideally approximating the diagonal line. The calibration curve for this model deviated minimally from the ideal curve (Figure 6). The Hosmer–Lemeshow test yielded *χ* ^2^ = 5.777, *p* = 0.672 (*p* > 0.05 indicates good model fit), and the Brier score was 0.164 (lower scores indicate more accurate predictions). Collectively, these results demonstrate good consistency between the model’s predicted values and actual observed values.

**FIGURE 7:**
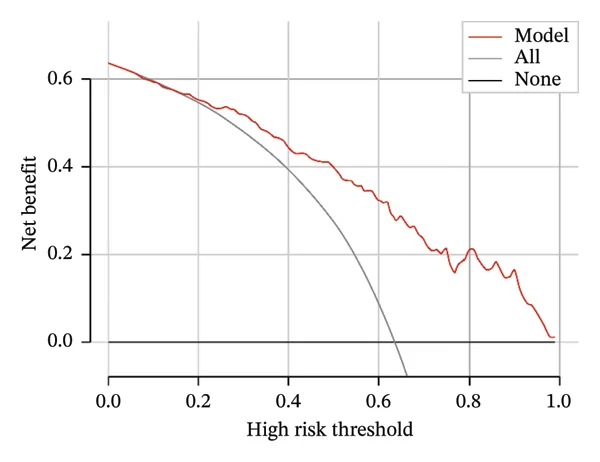
Decision curve analysis curve for the nomogram prediction model.

The rmda package was employed for decision curve analysis, evaluating the model’s clinical utility by calculating net benefit at different threshold probabilities (net benefit > 0 indicates that the model outperforms either full intervention or no intervention strategies). This model demonstrated positive net benefit across threshold probabilities ranging from 0.01 to 0.92, with a maximum net benefit of 0.633 observed at a risk threshold of 0.01. This indicates the model’s clinical utility across a broad spectrum of decision thresholds, while its application at lower risk thresholds maximizes clinical benefit (Figure 7).

## 4. Discussion

### 4.1. High Incidence of Compassion Fatigue Among Midwives

The findings of this study indicate that the prevalence of compassion fatigue among midwives was 91.86%, higher than that observed among nurses in other departments [8]. This suggests that midwives are more susceptible to compassion fatigue compared to other nursing staff, likely due to the high emotional involvement inherent in their work and the unique stressors specific to obstetrics. Our findings among midwives in western China align with those reported by Muliira et al. [15] among Ugandan midwives. This highlights that midwives in resource‐limited settings face heavier workloads compared to their counterparts in high‐income countries. Beyond shared stressors such as empathic engagement and traumatic birth events, midwives in low‐ and middle‐income countries may additionally confront structural challenges including inadequate infrastructure, suboptimal staffing levels, and insufficient psychological support resources [34].

Therefore, it is recommended that administrators introduce standardized assessment tools for midwives’ compassion fatigue. Through regular screening, high‐risk individuals can be accurately identified, providing a basis for preventive interventions. This shifts the focus of prevention and control from reactive measures to proactive prevention. In practice, this means implementing tiered interventions tailored to midwives based on assessment outcomes while prioritizing psychological support and resource allocation for those exhibiting higher levels of compassion fatigue. This approach enhances intervention efficiency, optimizes limited resources, and prevents wasteful, indiscriminate management practices. Additionally, healthcare institutions should enhance infrastructure and resource guarantees while optimizing human resource allocation. Workloads can be reduced through rational job placement and flexible scheduling. Concurrently, psychological support systems should be strengthened by establishing peer support groups, routine case review discussions, and other team‐based mutual aid mechanisms. Introducing professional psychological support will build a more resilient occupational health support system at the institutional level.

### 4.2. Analysis of Factors Influencing Compassion Fatigue Among Midwives

#### 4.2.1. Age Is a Significant Protective Factor Against Compassion Fatigue

The results of this study indicate that midwives aged ≥ 36 years exhibit a significantly lower risk of experiencing compassion fatigue compared to younger midwives aged 20–30 years (OR: 36–45 = 0.244, OR: > 45 = 0.082). This finding aligns with the results of Guzzon’s [35] cross‐sectional survey of Italian midwives, which indicated that secondary traumatic stress decreases with increasing age among midwives. As a core dimension of compassion fatigue, this finding reveals the protective role of age in mitigating compassion fatigue, particularly its traumatic components. By managing numerous complex clinical scenarios, senior midwives develop more mature emotional regulation strategies and psychological resilience. This enables them to maintain empathy while establishing clear emotional boundaries, preventing excessive involvement in mothers’ traumatic experiences, and effectively avoiding emotional resource depletion [36]. Moreover, extensive clinical experience enables senior midwives to more accurately recognize and anticipate potential stressors, reducing the unpredictability and perceived threat of their work. Therefore, managers should recognize that young midwives are a vulnerable group prone to compassion fatigue. In resource allocation and psychological support programs, they should be prioritized as targets for intervention and protection, receiving systematic professional skills training and guidance on building psychological resilience. Simultaneously, establishing mentorship or peer support systems can organize pairings between senior and junior midwives. Through sharing experiences and coping strategies, these arrangements provide emotional support and practical guidance to younger colleagues. Future research employing longitudinal designs could further clarify the causal relationship between age and compassion fatigue. It should also delve into the specific protective psychological mechanisms of midwives at different career stages, providing evidence‐based foundations for developing phased, targeted psychological support and human resource management strategies.

#### 4.2.2. Midwives Who Frequently Experience Insomnia Are at Higher Risk of Developing Compassion Fatigue

This study found that midwives’ sleep quality is an independent predictor of compassion fatigue, consistent with the findings of Qu et al.’s [16] investigation into factors influencing compassion fatigue among Chinese midwives. Midwives experiencing frequent insomnia showed a significantly increased risk of compassion fatigue (OR = 3.321, *p* < 0.05), indicating the significant impact of sleep issues on midwives’ occupational health. Sleep serves as a critical period for physiological and psychological restoration. High‐quality sleep aids in memory consolidation, emotional regulation, and energy replenishment. Midwives’ work typically involves prolonged continuous shifts characterized by high intensity, irregular day–night schedules, emergency delivery responses, and sustained mental tension. These factors readily disrupt normal circadian rhythms [37], leading to sleep disturbances and diminished sleep quality. Chronic sleep deprivation elevates stress hormones such as cortisol, impairing emotional regulation and cognitive control [38]. This diminishes midwives’ capacity to process emotions when confronting mothers’ emotional needs and pain, accelerating their descent into compassion fatigue. Therefore, nursing administrators should systematically evaluate and optimize shift schedules to prevent circadian rhythm disruption caused by frequent day–night shift rotations. Concurrently, they can regularly organize psychological sleep intervention programs such as sleep hygiene education, mindfulness‐based stress reduction, and cognitive‐behavioral therapy to enhance midwives’ ability to self‐regulate their sleep. Future research may further utilize objective sleep assessment tools and adopt a combined subjective‐objective methodology to more precisely elucidate the dynamic relationship between sleep and compassion fatigue. This would provide evidence‐based research support for developing occupational health protection programs tailored to midwives.

#### 4.2.3. Impact of Traumatic Events and Professional Recognition on Compassion Fatigue

Traumatic and supportive factors in the occupational environment jointly influence midwives’ compassion fatigue. The study findings indicate that the frequency of traumatic events is an independent risk factor for compassion fatigue among midwives (OR = 1.529, *p* < 0.05), while perceived recognition in the past month serves as a protective factor (OR = 0.363, *p* < 0.05). Midwives frequently encounter traumatic events in clinical practice, such as induced labor, postpartum hemorrhage, shoulder dystocia, and neonatal asphyxia [39, 40]. These events not only trigger acute stress responses but may also lead to long‐term psychological damage through cumulative trauma effects and empathy overload, thereby inducing compassion fatigue [41]. This mechanism of influence is highly similar across global midwifery populations. Professional recognition, as a vital psychosocial support resource, manifests as positive feedback and respect from patients’ families, colleagues, and management [6]. It not only enhances midwives’ sense of professional value and accomplishment but also effectively buffers the negative impacts and psychological shocks stemming from work stress [42], thereby mitigating the occurrence of compassion fatigue. Therefore, healthcare institutions should establish robust mechanisms for monitoring traumatic events and providing postevent support. This includes implementing structured incident reporting and debriefing systems, along with offering timely and accessible psychological counseling resources to help midwives process the emotional impact of high‐risk events. Simulation training and clear contingency plans can also enhance midwives’ skills and confidence in managing obstetric emergencies, thereby reducing psychological burdens from traumatic exposure at the source. Concurrently, a significant emphasis should be placed on cultivating a culture and mechanisms for professional recognition. Diverse, routine channels for acknowledgment and feedback should be established. Clinical administrators must proactively and openly affirm midwives’ professional contributions, fostering an organizational atmosphere of respect and support. This approach enhances their sense of professional purpose and psychological resilience.

#### 4.2.4. Moderating Role of Coping Strategies in Compassion Fatigue

Coping strategies represent a significant factor influencing midwives’ compassion fatigue. Findings indicate that positive coping (OR = 0.313, *p* < 0.001) serves as a significant protective factor, while negative coping (OR = 1.874, *p* = 0.003) constitutes a risk factor for compassion fatigue. This finding aligns with Zheng et al.’s [43] research on midwives’ post‐traumatic growth levels and influencing factors, underscoring coping styles’ pivotal role in post‐traumatic growth. Positive coping exerts a protective effect on compassion fatigue through resource acquisition and cognitive restructuring. It prompts midwives to proactively address problems; these positive behaviors not only enhance individuals’ sense of control over their work and reduce psychological stress but also facilitate the accumulation of new coping resources. The rational evaluation process within positive coping helps midwives reassess the meaning and impact of traumatic events, altering their cognitive and emotional responses. This enhances self‐efficacy and reduces empathic stress responses [44]. Conversely, negative coping depletes psychological resources and exacerbates compassion fatigue. When midwives employ negative coping strategies such as avoidance or self‐blame, they become prone to negative rumination. This rumination keeps individuals immersed in traumatic events and negative emotions, leading to excessive cognitive resource consumption and continuous depletion of emotional regulation capacity. Consequently, their ability to cope with stressors weakens, increasing susceptibility to vicarious trauma and emotional numbness [45], ultimately exacerbating symptoms of compassion fatigue. Therefore, coping strategies, as modifiable psychological and behavioral factors, should become key targets for compassion fatigue interventions. Nursing administrators should systematically integrate coping strategy training into midwives’ professional competency development and mental health support systems. Training should particularly emphasize cultivating rational assessment skills through cognitive‐behavioral training, stress management workshops, and scenario simulation exercises. This guides midwives in objectively analyzing events after emotional impact and adjusting irrational beliefs, thereby enhancing cognitive flexibility and emotional regulation abilities. Future research should further explore the specificity of coping approaches across cultural contexts and their mechanistic impact on compassion fatigue, while developing positive coping intervention programs tailored to local practice settings.

### 4.3. The Midwife Compassion Fatigue Risk Prediction Model Demonstrates Strong Predictive Value

Based on the results of multifactorial logistic regression analysis, this study identified six key predictors: age, sleep quality, frequency of traumatic events, perceived recognition in the past month, positive coping, and negative coping. To achieve intuitive visualization of these predictors, we constructed a nomogram model to depict the individualized probability of midwives experiencing compassion fatigue, which may assist managers in the preliminary identification of high‐risk individuals. Predictive efficacy of the model was systematically evaluated using multiple metrics: An AUC > 0.8 indicates good discriminative ability in distinguishing the occurrence of compassion fatigue. Internal validation results indicate that the model exhibits satisfactory stability, with its predicted probabilities exhibiting high consistency with actual observed outcomes. Decision curve analysis results suggest that this model may offer clinical utility across a range of threshold probabilities. Applying the model at lower risk thresholds may facilitate early identification of midwives at risk of compassion fatigue. Managers can implement targeted early interventions, prioritizing psychological support and workload adjustments for high‐risk individuals. These proactive measures may help mitigate the progression of compassion fatigue and could contribute to enhancing the stability and resilience of the midwifery workforce, potentially enabling responses to routine and emergency obstetric crises. The model is low‐cost, easy to implement, and highly accessible. It is applicable not only to the western Chinese region where this study was conducted but also holds promise as an efficient tool for resource‐constrained regions, pending external validation in diverse settings. It promotes the scientific and precision‐oriented development of global midwife mental health management. Managers could integrate this tool into routine occupational health assessments to screen midwives for compassion fatigue. Based on evaluation results, routine and targeted interventions can be implemented, which is crucial for maintaining the stability of the midwifery workforce and safeguarding maternal and infant health.

### 4.4. Limitations

This study has the following limitations. First, the sample originates from China’s western region, which is relatively homogeneous. However, the socioeconomic conditions in western China are comparable to those in low‐ and middle‐income countries globally, lending it a certain reference value. Second, while the study has preliminarily established a predictive model, it has not yet undergone external data validation. Given the modest EPV of approximately 8.4 and the use of internal validation only, there remains a potential risk of model overfitting, and the results should be interpreted with caution. Nevertheless, the model’s predictive efficacy was comprehensively evaluated using multiple indicators and validated internally, demonstrating sound predictive performance.

## 5. Implications for Nursing Management

This study reveals a high incidence of compassion fatigue among midwives. Managers should recognize the severity and urgency of this issue and prioritize it as a key focus in nursing management. The identified risk factors and the prediction model provide evidence‐based guidance for translating these findings into actionable management strategies.

To address psychological and trauma‐related risks, managers should establish accessible psychological support systems, including peer support groups and professional counseling services, to help midwives process emotional stress from clinical practice. For trauma‐informed support, healthcare institutions should implement structured incident reporting and debriefing systems following critical events (e.g., postpartum hemorrhage, shoulder dystocia, neonatal asphyxia) to facilitate timely emotional processing. Additionally, simulation training and clear contingency plans can enhance midwives’ confidence in managing obstetric emergencies, reducing psychological burden at the source. In parallel, managers should address modifiable behavioral and organizational factors. For midwives reporting poor sleep quality, shift schedules should be systematically evaluated and optimized to minimize frequent day–night rotations that disrupt circadian rhythms, and sleep hygiene programs or mindfulness‐based interventions can also be offered. To foster a culture of professional recognition, managers should implement regular, structured acknowledgment mechanisms—such as direct feedback from supervisors, peer‐nominated recognition, or public appreciation of clinical contributions—to reinforce midwives’ sense of professional value and accomplishment.

Building on these targeted interventions, the prediction model may support preliminary risk stratification for the implementation of tiered management approaches: Low‐risk individuals should receive routine mental health education and stress prevention courses (e.g., mindfulness training or team‐building activities) to enhance resilience; moderate‐risk individuals should undergo regular mental health screenings and receive personalized psychological support, such as short‐term counseling or peer support groups; high‐risk individuals require immediate, intensive interventions, including one‐on‐one psychological counseling, post‐traumatic stress support, job reassignments or rotational leave when necessary, and close monitoring. This tiered approach enhances intervention efficiency and ensures the precise allocation of psychological support resources. By integrating risk stratification with targeted psychological, organizational, and behavioral interventions, managers can systematically address both individual and systemic contributors to compassion fatigue, thereby improving midwives’ psychological well‐being, enhancing team stability, and safeguarding maternal and infant care quality.

The mental health status of midwives directly impacts care quality and patient safety. By systematically supporting midwives’ psychological well‐being, medical errors and declines in care quality resulting from compassion fatigue can be effectively minimized, thereby improving patient satisfaction and reducing the risk of medical disputes. Furthermore, grounded in the stress process theory, this model’s methodology and intervention logic can be extended to high‐pressure care units such as emergency departments and intensive‐care units. It provides a reference for preventing and managing compassion fatigue among nursing staff across different specialties, further expanding its broad value in optimizing healthcare human resource allocation and fostering organizational resilience.

## 6. Conclusions

This study, grounded in the stress process theory, found that 91.86% of midwives experienced compassion fatigue at any level; 63.6% had moderate to severe levels—the threshold considered clinically significant for the purpose of the prediction model. Age, sleep quality, perceived recognition in the past month, frequency of traumatic events, and positive/negative coping strategies were key factors influencing midwives’ compassion fatigue. Based on these predictors, a nomogram predictive model was constructed and validated. The model demonstrated good discrimination ability and calibration, offering nursing managers a practical, low‐cost tool for early identification of midwives at elevated risk of compassion fatigue and providing a reference for targeted preventive strategies.

## Author Contributions

Yian Zhan and Ying Li conceived and designed the study and conducted data collection. Yian Zhan and Shan Yang performed the statistical analysis and contributed to manuscript drafting. Ying Li critically revised and reviewed the manuscript for important intellectual content.

## Funding

This work was supported by the Gansu Provincial Science and Technology Plan Project (Natural Science Foundation Special Project) (Grant No. 26JRRA328).

## Disclosure

All authors reviewed and approved the final manuscript.

## Ethics Statement

This study was approved by the Institutional Review Board of the authors’ affiliated university (No. LZUHLXY20250053).

## Consent

All participants provided written informed consent after reviewing the study information, which described the study objectives, procedures, voluntary nature of participation, protection of anonymity, and data management.

## Conflicts of Interest

The authors declare no conflicts of interest.

## Supporting Information

Additional supporting information can be found online in the Supporting Information section.

## Supporting information


**Supporting Information** The following supporting information is available with this article: Supplementary File 1: TRIPOD checklist for prediction model studies. This file provides the completed checklist demonstrating adherence to the TRIPOD reporting guidelines.

## Data Availability

Data are available on request from the authors.

## Appendix A

**Table A1:** General characteristics of midwives and univariate analysis results for compassion fatigue (full version; *n* = 209).

Variable		Level of compassion fatigue		Statistics	*p* value
		No or mild	Moderate and above		
Age, *n* (%)	20–30 years	14 (18.4)	45 (33.8)	3.693^2^	< 0.001^∗^
	31–35 years	22 (28.9)	53 (39.8)		
	36–45 years	33 (43.4)	30 (22.6)		
	> 45 years	7 (9.2)	5 (3.8)		

Marital status, *n* (%)	Married	69 (90.8)	110 (82.7)	2.570^1^	0.109
	Unmarried or divorced	7 (9.2)	23 (17.3)		

Years of employment, *n* (%)	1–5 years	15 (19.7)	33 (24.8)	2.236^2^	0.025^∗^
	6–10 years	22 (28.9)	50 (37.6)		
	11–15 years	16 (21.1)	32 (24.1)		
	> 15 years	23 (30.3)	18 (13.5)		

Educational level, *n* (%)	Diploma or associate degree	16 (21.1)	21 (15.8)	0.957^2^	0.339
	Bachelor’s degree or higher	60 (78.9)	112 (84.2)		

Professional title, *n* (%)	Junior nurse	3 (3.9)	15 (11.3)	2.317^2^	0.021^∗^
	Staff nurse	29 (38.2)	61 (45.9)		
	Charge nurse	40 (52.6)	52 (39.1)		
	Nurse manager or higher	4 (5.3)	5 (3.8)		

Monthly income, *n* (%)	< ¥3000	10 (13.2)	25 (18.8)	1.782^2^	0.075
	¥3000–4999	28 (36.8)	57 (42.9)		
	¥5000–6999	20 (26.3)	30 (22.6)		
	¥≥¥7000	18 (23.7)	21 (15.8)		

Form of employment, *n* (%)	Contract worker	50 (65.8)	85 (63.9)	1.540^1^	0.463
	Personnel agency nurse	13 (17.1)	31 (23.3)		
	Staff nurse	13 (17.1)	17 (12.8)		

Sleep quality, *n* (%)	Normal	18 (23.7)	12 (9.0)	3.750^2^	< 0.001^∗^
	Occasional insomnia	38 (50.0)	55 (41.4)		
	Frequent insomnia	20 (26.3)	66 (49.6)		

Number of children, *n* (%)	0	13 (17.1)	32 (24.1)	2.288^2^	0.022^∗^
	1	26 (34.2)	59 (44.4)		
	2 or more	37 (48.7)	42 (31.6)		

Children’s status (children in junior high school or above), *n* (%)	No	56 (73.7)	115 (86.5)	5.312^1^	0.021^∗^
	Yes	20 (26.3)	18 (13.5)		

Number of adverse deliveries (since joining the hospital), *n* (%)	0	36 (47.4)	50 (37.6)	0.837^2^	0.402
	1–2 times	16 (21.1)	44 (33.1)		
	3–4 times	9 (11.8)	10 (7.5)		
	≥ 5 times	15 (19.7)	29 (21.8)		

Night shift frequency, *n* (%)	0 times/week	8 (10.5)	13 (9.8)	1.130^2^	0.259
	1–2 times/week	52 (68.4)	81 (60.9)		
	≥ 3 times/week	16 (21.1)	39 (29.3)		

Job satisfaction with compensation, *n* (%)	Satisfied	39 (51.3)	39 (29.3)	2.935^2^	0.003^∗^
	Normal	29 (38.2)	73 (54.9)		
	Unsatisfied	8 (10.5)	21 (15.8)		

Workload, *n* (%)	Excessive	24 (31.6)	62 (46.6)	1.806^2^	0.071
	Moderate	52 (68.4)	67 (50.4)		
	Light	0 (0.0)	4 (3.0)		

Shift duration, *n* (%)	≤ 8 h	34 (44.7)	43 (32.3)	1.416^2^	0.157
	8.5–11 h	20 (26.3)	46 (34.6)		
	> 11 h	22 (28.9)	44 (33.1)		

Competitive pressure, *n* (%)	Excessive	29 (38.2)	54 (40.6)	0.512^2^	0.608
	Moderate	43 (56.6)	75 (56.4)		
	Light	4 (5.3)	4 (3.0)		

Teamwork satisfaction, *n* (%)	Satisfied	47 (61.8)	47 (35.3)	3.600^2^	< 0.001^∗^
	Normal	24 (31.6)	69 (51.9)		
	Unsatisfied	5 (6.6)	17 (12.8)		

Annual leave entitlement, *n* (%)	≤ 4 days	7 (9.2)	25 (18.8)	2.868^2^	0.004^∗^
	5–9 days	34 (44.7)	71 (53.4)		
	≥ 10 days	35 (46.1)	37 (27.8)		

Work atmosphere satisfaction, *n* (%)	Satisfied	58 (76.3)	73 (54.9)	3.180^2^	0.001^∗^
	Normal	18 (23.7)	55 (41.4)		
	Unsatisfied	0 (0.0)	5 (3.8)		

Perceived recognition in the past month, *n* (%)	No	11 (14.5)	49 (36.8)	11.823^1^	< 0.001^∗^
	Yes	65 (85.5)	84 (63.2)		

Frequency of traumatic events, mean ± SD		8.05 ± 3.98	9.74 ± 4.71	2.662^2^	0.008^∗^

Impact of traumatic events, mean ± SD		30.01 ± 9.38	30.90 ± 8.92	0.671^2^	0.503

Social support, mean ± SD		44.75 ± 8.09	39.36 ± 7.84	4.724^3^	< 0.001^∗^

Psychological resilience, mean ± SD		27.50 ± 6.81	20.79 ± 7.71	5.916^2^	< 0.001^∗^

Positive coping strategies, mean ± SD		25.70 ± 6.72	19.82 ± 7.49	5.262^2^	< 0.001^∗^

Negative coping strategies, mean ± SD		11.87 ± 5.20	11.78 ± 4.85	0.239^2^	0.811

Abbreviation: SD, standard deviation.

^1^ *χ* ^2^.

^2^Z.

^3^t.

^∗^ *p* < 0.05.

**Table A2:** Comparative analysis of logistic regression models for risk factors of compassion fatigue among midwives.

Number	Variable inclusion method	Variables included in the model	Omnibus tests of model coefficients	Hosmer–Lemeshow test
1	Forward (single‐factor significant variables)	Age, sleep quality, psychological resilience, and social support	*χ* ^2^ = 59.042, *p* < 0.001 −2 log likelihood = 214.949 Cox–Snell *R* ^2^ = 0.246 Nagelkerke *R* ^2^ = 0.337	*R* ^2^ = 15.732 *p* = 0.046

2	Backward (single‐factor significant variables)	Age, perceived recognition, frequency of traumatic events, positive coping strategies, and psychological resilience	*χ* ^2^ = 62.074, *p* < 0.001 −2 log likelihood = 211.917 Cox–Snell *R* ^2^ = 0.257 Nagelkerke *R* ^2^ = 0.352	*R* ^2^ = 5.806 *p* = 0.669

3	Forward (all variables)	Age, sleep quality, social support, and psychological resilience	*χ* ^2^ = 59.042, *p* < 0.001 −2 log likelihood = 214.949 Cox–Snell *R* ^2^ = 0.246 Nagelkerke *R* ^2^ = 0.337	*R* ^2^ = 15.732 *p* = 0.046

4	Backward (all variables)	Age, professional title, sleep quality, perceived recognition, frequency of traumatic events, positive coping strategies, and negative coping strategies	*χ* ^2^ = 75.299, *p* < 0.001 −2 log likelihood = 198.693 Cox–Snell *R* ^2^ = 0.303 Nagelkerke *R* ^2^ = 0.414	*R* ^2^ = 7.540 *p* = 0.480

**Table A3:** Variable assignment.

Variable	Assignment method	Meaning of dummy variables
Compassion fatigue	No or mild = 0; no or mild = 1	Binary categorical variable; no dummy variables required
Age	20–30 years old (0, 0, 0, 0); 31–35 years old (0, 1, 0, 0); 36–45 years old (0, 0, 1, 0); > 45 years old (0, 0, 0, 1)	*Z*_1_ = 31–35 years old; *Z*_2_ = 36–45 years old; *Z*_3_ = > 45 years old
Marital status	Unmarried or divorced = 0; married = 1	Binary categorical variable, no dummy variables required
Years of employment	1–5 years (0, 0, 0, 0); 6–10 years (0, 1, 0, 0); 11–15 years (0, 0, 1, 0); > 15 years (0, 0, 0, 1)	*Z*_1_ = 6–10 years; *Z*_2_ = 11–15 years; *Z*_3_ = > 15 years
Hospital level	Large general hospital (0, 0, 0, 0); large specialized hospital (0, 1, 0, 0); medium‐sized general hospital (0, 0, 1, 0); medium‐sized specialized hospital (0, 0, 0, 1)	*Z*_1_ = large specialized hospital; *Z*_2_ = medium‐sized general hospital; *Z*_3_ = medium‐sized specialized hospital
Educational level	Diploma or associate degree = 0; bachelor’s degree or higher = 1	Binary categorical variable; no dummy variables required
Professional title	Junior nurse (0, 0, 0, 0); staff nurse (0, 1, 0, 0); charge nurse (0, 0, 1, 0); nurse manager or higher (0, 0, 0, 1)	*Z*_1_ = staff nurse; *Z*_2_ = charge nurse; *Z*_3_ = nurse manager or higher
Monthly income	< ¥3000 (0, 0, 0, 0); ¥3000∼4999 (0, 1, 0, 0); ¥5000∼6999 (0, 0, 1, 0); ¥≥ 7000 (0, 0, 0, 1)	*Z*_1_ = ¥3000∼4999; *Z*_2_ = ¥5000∼6999; *Z*_3_ = ≥ ¥7000
Form of employment	Contract worker (0, 0, 0); personnel agency nurse (0, 1, 0); staff nurse (0, 0, 1)	*Z*_1_ = personnel agency nurse; *Z*_2_ = staff nurse
Sleep quality	Normal (0, 0, 0); occasional insomnia (0, 1, 0); frequent insomnia (0, 0, 1)	*Z*_1_ = occasional insomnia; *Z*_2_ = frequent insomnia
Number of children	0 (0, 0, 0); 1 (0, 1, 0); 2 or more (0, 0, 1)	*Z*_1_ = 1; *Z*_2_ = 2 or more
Children’s status	No = 0; yes = 1	Binary categorical variable; no dummy variables required
Number of adverse deliveries	0 (0, 0, 0, 0); 1–2 times (0, 1, 0, 0); 3–4 times (0, 0, 1, 0); ≥ 5 times (0, 0, 0, 1)	*Z*_1_ = 1–2 times; *Z*_2_ = 3–4 times; *Z*_3_ = ≥ 5 times
Night shift frequency	0 (0, 0, 0); 1–2 times/week (0, 1, 0); ≥ 3 times/week (0, 0, 1)	*Z*_1_ = 1–2 times/week; *Z*_2_ = ≥ 3 times/week
Job satisfaction with compensation	Satisfied (0, 0, 0); Normal (0, 1, 0); Unsatisfied (0, 0, 1)	*Z*_1_ = Normal; *Z*_2_ = Unsatisfied
Workload	Excessive (0, 0, 0); moderate (0, 1, 0); light (0, 0, 1)	*Z*_1_ = moderate; *Z*_2_ = light
Shift duration	≤ 8 h (0, 0, 0); 8.5∼11 h (0, 1, 0); > 11 h (0, 0, 1)	*Z*_1_ = 8.5∼11 h; *Z*_2_ = > 11 h
Competitive pressure	Excessive (0, 0, 0); moderate (0, 1, 0); light (0, 0, 1)	*Z*_1_ = moderate; *Z*_2_ = light
Teamwork satisfaction	Satisfied (0, 0, 0); normal (0, 1, 0); unsatisfied (0, 0, 1)	*Z*_1_ = normal; *Z*_2_ = unsatisfied
Annual leave entitlement	≤ 4 days (0, 0, 0); 5∼9 days (0, 1, 0); ≥ 10 days (0, 0, 1)	*Z*_1_ = 5∼9 days; *Z*_2_ = ≥ 10 days
Work atmosphere satisfaction	Satisfied (0, 0, 0); normal (0, 1, 0); unsatisfied (0, 0, 1)	*Z*_1_ = normal; *Z*_2_ = unsatisfied
Perceived recognition in the past month	No = 0; yes = 1	Binary categorical variable, no dummy variables required
Frequency of traumatic events	Original value *Z*‐score standardized and substituted	Continuous variables; no dummy variables required
Impact of traumatic events	Original value *Z*‐score standardized and substituted	Continuous variables; no dummy variables required
Social support	Original value *Z*‐score standardized and substituted	Continuous variables; no dummy variables required
Psychological resilience	Original value *Z*‐score standardized and substituted	Continuous variables; no dummy variables required
Positive coping strategies	Original value *Z*‐score standardized and substituted	Continuous variables; no dummy variables required
Negative coping strategies	Original value *Z*‐score standardized and substituted	Continuous variables; no dummy variables required

**Table A4:** Scores for each variable.

Variable	Level/raw score	Corresponding score (points)
Age	> 45 years old	0
	36–45 years old	21.59
	31–35 years old	45.85
	20–30 years old	49.58

Sleep quality	Normal	0
	Occasional insomnia	12.52
	Frequent insomnia	23.81

Perceived recognition in the past month	Yes	0
	No	20.10

Frequency of traumatic events	0	0
	2	3.73
	4	7.46
	6	11.19
	8	14.92
	10	18.65
	12	22.38
	14	26.11
	16	29.84
	18	33.57
	20	37.30
	22	41.03

Positive coping strategies	24	0
	22	5.95
	20	11.90
	18	17.86
	16	23.81
	14	29.76
	12	35.71
	10	41.67
	8	47.62
	6	53.57
	4	59.52
	2	65.48
	0	71.43

Negative coping strategies	0	0
	5	12.5
	10	25
	15	37.5
	20	50
	25	62.5
	30	75
	35	87.5
	40	100

*Note:* Each variable is scored as follows: (1) Age: > 45 years scores 0 points, 36–45 years scores 21.59 points, 31–35 years scores 45.85 points, and 20–30 years scores 49.58 points. (2) Sleep quality: Normal scores 0 points, occasional insomnia scores 12.52 points, and frequent insomnia scores 23.81 points. (3) Perceived recognition in the past month: Yes scores 0 points, and no scores 20.10 points. (4) Frequency of traumatic events: The lowest score [0] corresponds to 0 points. For every 2‐point increase in the raw score, this variable increases by 3.73 points. (5) Positive coping: The maximum score [24] corresponds to 0 points; for every 2‐point decrease in raw score, this item increases by 5.95 points. (6) Negative coping: The minimum score [0] corresponds to 0 points; for every 5‐point increase, this item increases by 12.5 points.

**Table A5:** Predicted probability corresponding to the total score.

Predicted probability	Total score (points)
0.05	1.25
0.1	18.06
0.2	36.31
0.3	48.44
0.4	58.38
0.5	67.50
0.6	76.62
0.7	86.56
0.8	98.69
0.9	116.94
0.95	133.75
0.99	170.89

**Table A6:** Relationship between the bootstrap sampling frequency and corrected area under the curve (AUC).

	1	2	3	4	5	6
BootReps	200	500	800	1000	1200	1500
AUC	0.784	0.787	0.786	0.786	0.785	0.786

*Note:* BootReps: Bootstrap replications (number of bootstrap resampling iterations).
